# *Palaeospondylus* as a primitive hagfish

**DOI:** 10.1186/s40851-016-0057-0

**Published:** 2016-09-08

**Authors:** Tatsuya Hirasawa, Yasuhiro Oisi, Shigeru Kuratani

**Affiliations:** 1 Evolutionary Morphology Laboratory, RIKEN, 2-2-3 Minatojima-minami, Chuo-ku, Kobe, Hyogo 650-0047 Japan; 2 Development and Function of Inhibitory Neural Circuits, Max Planck Florida Institute for Neuroscience, Jupiter, FL 33458 USA

**Keywords:** Cyclostomes, Devonian, Fossil taxa, Hagfish, Homology, *Palaeospondylus*

## Abstract

**Background:**

The taxonomic position of the Middle Devonian fish-like animal *Palaeospondylus* has remained enigmatic, due mainly to the inability to identify homologous cranial elements. This animal has been classified into nearly all of the major vertebrate taxa over a century of heuristic taxonomic research, despite the lack of conclusive morphological evidence.

**Results:**

Here we report the first comparative morphological analysis of hagfish embryos and *Palaeospondylus*, and a hitherto overlooked resemblance in the chondrocranial elements of these animals; *i.e*., congruence in the arrangement of the nasal capsule, neurocranium and mandibular arch-derived velar bar. The large ventral skeletal complex of *Palaeospondylus* is identified as a cyclostome-specific lingual apparatus. Importantly, the overall morphological pattern of the *Palaeospondylus* cranium coincides well with the cyclostome pattern of craniofacial development, which is not shared with that of crown gnathostomes. Previously, the presence of the vertebral column in *Palaeospondylus* made its assignment problematic, but the recent identification of this vertebral element in hagfish is consistent with an affinity between this group and *Palaeospondylus*.

**Conclusion:**

These lines of evidence support the hagfish affinity of *Palaeospondylus*. Moreover, based on the less specialized features in its cranial morphology, we conclude that *Palaeospondylus* is likely a stem hagfish.

**Electronic supplementary material:**

The online version of this article (doi:10.1186/s40851-016-0057-0) contains supplementary material, which is available to authorized users.

## Background

**Fig. 1:**
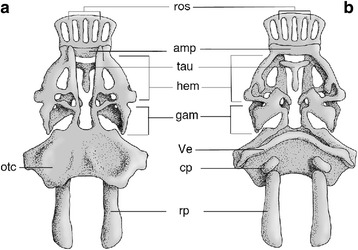
Cranial skeletons of *Palaeospondylus*. **a**,**b** Restoration of *Palaeospondylus gunni* cranial skeleton in dorsal (**a**) and ventral (**b**) views. amp, ampyx; cp, caudal plate; gam, gammation; hem, hemidome; otc, otic capsule; ros, rostralia; rp, rostral plate; tau, tauidion; Ve, V-shaped element

*Palaeospondylus gunni* [1] from the Middle Devonian of Scotland (Fig. 1, Additional file 1: Figure S1, Additional file 2: Figure S2) has long been an enigmatic fossil in vertebrate palaeontology [1–11]. Over the past 125 years, attempts have been made to classify this fossil as a cyclostome [1, 6, 8], frog tadpole [2], lungfish larva [3, 7, 10], holocephalan [4], elasmobranch [5], placoderm [9], and even a secondarily boneless osteichthyan [11]; however, none of these assignments has been supported by conclusive evidence [11–13]. This problem has been attributed to an inability to homologize its skeletal elements [14], and previous hypotheses have never successfully explained its anatomical configuration. Indeed, the arrangement of skeletal elements of *Palaeospondylus* has never yet been integrally compared to any of the actual ontogenetic stages of certain taxa.

Morphological homology [15–17] is not recognized *a priori*, but is adopted when the topographical relationships among a series of comparable body parts are found consistently, through heuristic search, in a certain clade. Logically, cladistic analyses cannot be applied prior to the identification of homologies [15, 18], and it is difficult to undertake a dynamic homology approach [19] when analyzing unintelligible morphological data. Unlike molecular-based phylogenetic analyses utilizing discrete characters (base or amino acid sequences), most morphology-based analyses require anatomical realization for alignments of corresponding units, that is, character codings. For yet-unassigned fossil taxa, such as *Palaeospondylus*, the discovery of the most parsimonious set of homologous body parts is the only solution to the classification problem [14].

Until recently, a lack of morphological data in hagfish, developmental data in particular, has prevented the heuristic search for homologies in entire vertebrate groups. Recent studies of the embryonic development of the hagfish [20–23] have gone some way to addressing this deficiency. Analyses of the embryonic development of the hagfish resolved the disparity in cranial morphology of vertebrates [22, 24], and for the first time, made it possible to compare the cranial skeleton of *Palaeospondylus* with those of a wide range of vertebrate groups. Between the cyclostomes and crown gnathostomes (jawed gnathostomes), there is a profound disparity in craniofacial pattern, which originates from the difference in arrangement of craniofacial primordia during embryonic development [22, 24]. Importantly, although the cyclostome pattern likely represents the ancestral condition for the vertebrates as an entire group, crown gnathostome development does not follow the cyclostome pattern [22, 24]. Based on this disparity, which is due to the complete loss of some vertebrate plesiomorphies in the crown gnathostomes, the possibility of the crown gnathostome affinity of a certain taxon can be eliminated by identifying a set of homologous elements seen in the extant cyclostomes.

From the perspective of morphological disparity, classification of the enigmatic taxon *Palaeospondylus* into a higher taxonomic group within the vertebrates is achievable through the identification of congruence in arrangement of a suite of homologous elements. Attempts to identify homologous elements between *Palaeospondylus* and various vertebrate groups have been made, but the hagfish embryo has remained unexamined. In this study, we compared embryos of the inshore hagfish *Eptatretus burgeri* and *Palaeospondylus*, focusing on phylogenetically informative features, and discovered homologous skeletal parts between them.

## Methods

### Observation of hagfish embryos

Embryos of the inshore hagfish *Eptatretus burgeri* were collected according to the developmental stage table of [25], as described previously [20]. In this study, we reexamined three-dimensional models reconstructed from the serial histological sections of the hagfish embryos previously [22, 23].

### Observation of *Palaeospondylus* fossils

Specimens of *Palaeospondylus* were observed using a stereomicroscope (M7A, Wild, Heerbrugg, Switzerland) at the American Museum of Natural History (AMNH), New York, USA. Images were taken using a digital camera (E-PL6, Olympus Corp., Tokyo, Japan) mounted on the stereomicroscope via an adaptor (NY-1S, Microscope Network Co., Ltd., Saitama, Japan). The specimens of *Palaeospondylus* expose either dorsal or ventral aspects of their cranial skeletons on the slabs. Based on positional relationship to vertebral column, which lay dorsally in the live body, we identified dorsal and ventral views of the *Palaeospondylus* cranial skeletons on the slabs.

## Results

### Key features in the embryonic development of the modern hagfish *Eptatretus*

**Fig. 2:**
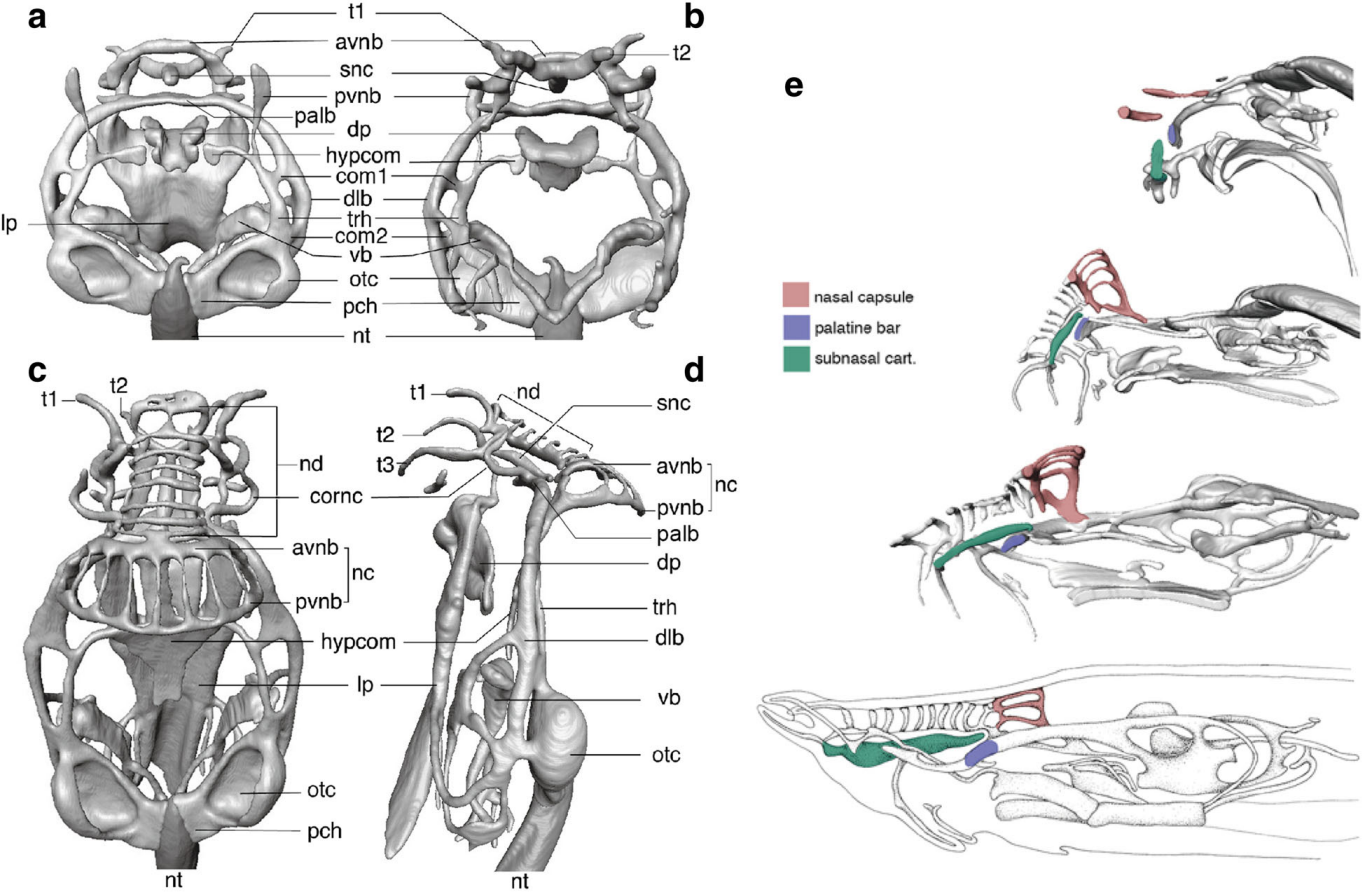
Cranial skeletons of the *Eptatretus* embryo. **a** *Eptatretus burgeri* embryo at stage 53 in dorsal view. **b** *E. burgeri* embryo at stage 53 in ventral view. **c** *E. burgeri* embryo at stage 60 in dorsal view. **d** *E. burgeri* embryo at stage 60 in left lateral view. **e** Relative growth of the developing hagfish chondrocrania. From top to bottom, stage 53, stage 60, prehatching and adult hagfish crania. Note, due to the rostral growth of the snout by addition of nasal duct cartilage and extension of subnasal cartilage (*green*), that the position of the nasal capsule (*red*) shifts relatively caudal through development. The palatine bar, or the transverse commissure on the rostral tip of the dorsal longitudinal bar, is colored blue for the reference. avnb, anterior vertical nasal bar; com1, 2, commissures of dlb; cornc, cornual cartilage; dlb, dorsal longitudinal bar; dp, dental plate; hypcom, hypophyseal commissure; lp, lingual plate; nc, nasal capsule; nd, nasal duct cartilage; nt, notochord; otc, otic capsule; palb, palatine bar; pch, parachordal; pvnb, posterior vertical nasal bar; rp, rostral plate; snc, subnasal cartilage; t1-3, cartilaginous support for tentacles; trh, trabecula of hagfish; vb, velar bar

In *E. burgeri* embryos at stages 53–60 (Fig. 2a–d), the neurocranium consists of two pairs of longitudinal bars with transverse commissures and processes, rostrally attached to the nasal capsule. Caudally, the longitudinal bars are contiguous to the otic capsule, which continues medially to the parachordal that surrounds the rostral part of the notochord (Fig. 2a, b). From the boundary between the neurocranium and otic capsule, a pair of velar bars grows ventromedially, and later, by stage 53, becoming fused at the median plane to form a V-shaped element. The dental plate of the oral apparatus is located below the neurocranium, associated with the rostral part of the lingual plate that supports the muscles of the cyclostome ‘tongue’ [22]. The topographical relationships between these cranial elements in hagfish embryos continue to change over the course of development [23]. Prior to hatching, the nasal capsule grows cartilages supporting the rostrally extended nasal duct (Fig. 2e). Simultaneously, cartilages in the oronasal septum extend rostrally, and supporting cartilages arise within tentacles, features that are specific to adult individuals in all known hagfish species. These changes result in a caudad shift in the relative position of the nasal capsule in the cranium.

### Homologies between *Eptatretus* and *Palaeospondylus*

**Fig. 3:**
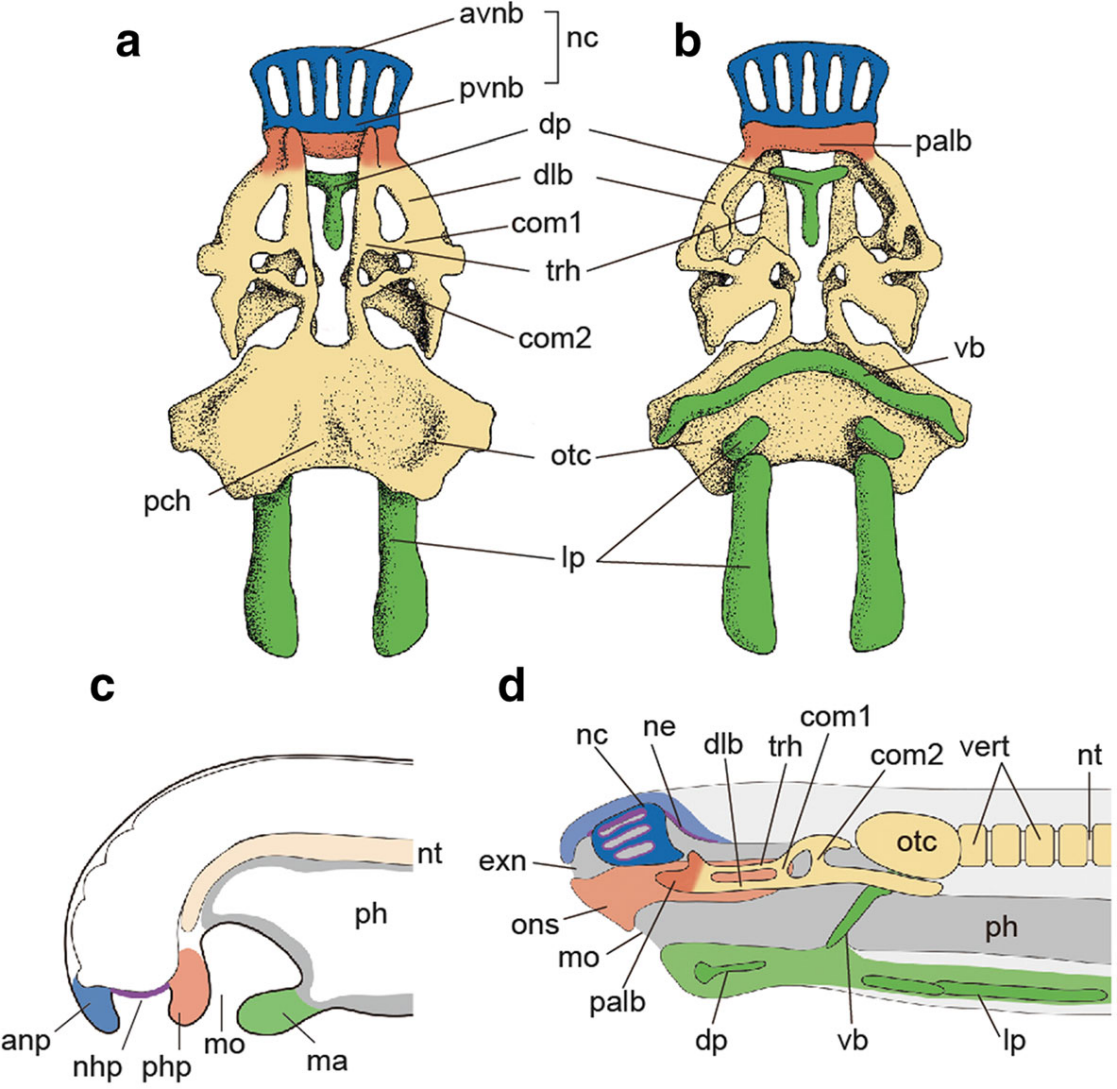
Developmental origins of *Palaeospondylus* cranial skeleton. **a**,**b** Diagrammatic anatomy of the cranial skeleton in dorsal (**a**) and ventral (**b**) views. **c** Cyclostome pattern in embryonic development of lamprey, hagfish, and also of *Palaeospondylus*. **d** Hypothetical developmental configuration of *Palaeospondylus* derived from the cyclostome craniofacial pattern. anp, anterior nasal process and its derivatives; ma, mandibular arch; mo, mouth; ne, nasal epithelium; nhp, nasohypophyseal plate; ph, pharynx; php, post-hypophyseal process and its derivatives; vert, vertebra. For other abbreviations, see Fig. 2

As in cyclostomes, the cranium of *Palaeospondylus* is generally believed to consist of endoskeletal elements [5, 8, 9]. In many *Palaeospondylus* specimens, the vertebral column is preserved, and it is generally accepted that the massive skeletal element at the level of the first vertebral element represents the otic capsule [3–9] (Figs. 1 and 3a, b, Additional file 1: Figure S1, Additional file 2: Figure S2). We use this feature as a landmark in our identification of other skeletal elements in this species. Our observations (Additional file 1: Figure S1) and a review of the literature [5, 8, 26] (Additional file 2: Figure S2) indicate that *Palaeospondylus* possessed a neurocranium comparable to that of the hagfish embryo, in terms of the possession of two pairs of longitudinal bars (the dorsal longitudinal bar and hagfish trabecula), the palatine bar, and other commissures connecting these bars (Figs. 2 and 3a, b). Rostrally, several (presumably six) longitudinal bars run in parallel (‘rostralia’ in the previous description [5]; Fig. 1a, Additional file 1: Figure S1, Additional file 2: Figure S2). These bars are contiguous caudally with a transverse bar and are expanded towards the rostral end to contact with adjacent elements (Fig. 1a, Additional file 1: Figure S1, Additional file 2: Figure S2), forming a cage-shaped structure [5, 9]. The shape and position of this structure, and in particular the presence of both the anterior and posterior transverse bars, show strong morphological similarities with those of the nasal capsule in the hagfish embryo (Fig. 3a). The nasal capsule of *Palaeospondylus* was previously identified at a lateral part of the neurocranium (‘hemidome’) [10], but in that model, the other neurocranial elements, including the trabeculae (in this case, the trabecula of crown gnathostomes), could not be identified. In contrast, the homologization between *Palaeospondylus* and the hagfish embryo described above succeeds in identifying each skeletal element at a comparable position.

The ventral V-shaped element in *Palaeospondylus* (Fig. 1b, Additional file 1: Figure S1, Additional file 2: Figure S2) resembles the velar bar of the hagfish embryo, as both elements are suspended ventrally at the level of the junction between the neurocranium and otic capsule (Fig. 3b). The midline of the V-shaped element of *Palaeospondylus* is mostly unfused, but this does not contradict the homology as, in the hagfish embryo, the velar bar develops from paired cartilages and later becomes fused at the median plane by stage 53. In some previous studies, this element has been identified as the gnathostome ceratohyal [8, 9]; however, this fails to explain the apparent absence of more posterior branchial arches in *Palaeospondylus*. The ventral, median T-shaped skeletal element in *Palaeospondylus*, often called the ‘tauidion’ [5] (Fig. 1b), may occur distant from the neurocranium [5, 8, 9, 26] (Additional file 2: Figure S2), and we suggest that it is comparable to the hagfish dental plate (Figs. 2b, d and 3b, Additional file 2: Figure S2). In the previous gnathostome hypothesis, this ‘tauidion’ has been analogized to the gnathostome vomer [10], but that identification is less plausible, as the ‘tauidion’ was independent of the other cranial elements. The absence of dermal bones in *Palaeospondylus* [11] also argues against the interpretation of the ‘tauidion’ as the gnathostome vomer, which is a dermal bone.

Two paired skeletal plates cover the ventral aspect of the caudal half of *Palaeospondylus* cranium (Fig. 1). The rostral pair is located at the level of the otic capsule, and the larger caudal pair covers the ventral aspect at the levels of the caudal half of the otic capsule and the rostral vertebral elements (Fig. 1b). These plates likely represent the lingual apparatus of the hagfish (Fig. 3b), as proposed also by Bulman [8]. These plates were later identified as lungfish-specific cranial ribs (occipital ribs) [10]. This view, however, is not consistent with the absence of the cranial rib in larval lungfishes [27, 28].

## Discussion

The history of studies on *Palaeospondylus* [1–10] constitutes a heuristic search for homologous body parts, a task that we suggest may be completed with the present study, in which it was finally compared with the hagfish embryo. Previous studies comparing *Palaeospondylus* with hagfishes relied solely on adult morphology [8]. Importantly, it has recently been shown that adult hagfish species possess vestigial vertebral elements [21]. The presence of a vertebral column composed of cylindrical vertebrae in *Palaeospondylus* (Additional file 1: Figure S1), which simply sheathed the notochord [5, 8], thus does not necessarily preclude its affinity with the modern hagfish. Our comparative analysis of *Palaeospondylus* and the hagfish embryo, revealing previously unrecognized topographical relationships between skeletal elements specific to these taxa, was made possible by the introduction of modern techniques to the study of hagfish embryonic development in recent years [20–23, 29].

According to comparative developmental biology two mutually exclusive systems of craniofacial anatomical configuration are present in vertebrates [22, 24]. In both, during embryonic development, the rostral portion of the cephalic crest-derived ectomesenchyme (trigeminal crest cells) initially follows a common distribution pattern, comprising the pre- and postoptic crest cells and mandibular arch crest cells (up to early pharyngula stage) [30]. However, the difference between the cyclostomes and crown gnathostomes later becomes conspicuous (Additional file 3: Table S1) in association with differences in placode distribution [22, 31]. In cyclostomes, the craniofacial morphology develops from the mandibular arch (MA), post-hypophyseal process (PHP), and anterior nasal process (ANP) [24, 32]. In crown gnathostomes, the embryonic head consists of lateral and medial nasal prominences (LNP, MNP), postoptic ectomesenchyme (PO), maxillary and mandibular processes (MX, MN) [24, 32]. Given that the craniofacial pattern of stem gnathostomes (osteostracans and galeaspids) conforms with the cyclostome pattern [22], the cyclostome pattern (involving MA, PHP, and ANP) thus represents the ancestral developmental pattern for the total-group vertebrates, whereas the crown gnathostome pattern (involving LNP, MNP, PO, MX, and MN) represents a derived condition. However, the craniofacial development of crown gnathostomes does not, even transiently, recapitulate the cyclostome pattern at any developmental stage [22, 24].

The *Palaeospondylus* cranium is congruent with the cyclostome craniofacial pattern described above (Fig. 3). In this comparison, the nasal capsule, subnasal rostral cranium (palatine bar, and rostral part of the dorsal longitudinal bar and trabecula), and dental and lingual plates of the *Palaeospondylus* are reconstructed to develop from the ANP, PHP and MA of the cyclostome pattern, respectively (Fig. 3c,d; Additional file 4: Table S2). The inferred positions of the nasal epithelium and adenohypophysis in *Palaeospondylus* are consistent with this scheme (Fig. 3d). Contrastingly the *Palaeospondylus* cranium cannot be derived from the crown gnathostome pattern, which involves a dorsoventrally bifurcated mandibular arch, separate paired nostrils, and adenohypophysis. A previous hypothesis that suggested a Devonian lungfish affinity of *Palaeospondylus* [10] was flawed in that it did not account for the neurocranial element developed medially to MX.

More detailed homology relationship of skeletal elements can be established between *Palaeospondylus* and the hagfish embryo at specific developmental stages, but not with the lamprey skeletal elements at any developmental stage. The cage-shaped nasal capsule, two separated longitudinal bars (the dorsal longitudinal bar and trabecula), velar bar, and large lingual plates (Fig. 3a, b) are seen exclusively in the hagfish embryo and *Palaeospondylus*, and the topographical arrangement of these homologous elements is consistent. Based on these synapomorphies, it is parsimonious that *Palaeospondylus* was related to hagfishes.

A comparison of the rostral portion of the neurocranium among vertebrates further underscores the congruence between *Palaeospondylus* and the hagfish. There is a morphological disparity among the crown gnathostomes, lamprey and hagfish, which results from the difference in ‘trabeculae’ within embryonic heads; in fact, the term ‘trabecula’ does not designate the same body part among the crown gnathostomes, lamprey and hagfish. The neurocranium of the crown gnathostomes is heterogeneous in cell lineage [30, 33]; the rostral part, or trabecula, develops from the cephalic neural crest cells [34–38], while the caudal part, or parachordals, from the mesoderm [33]. In the cyclostomes, a large part of the rostral portion of the neurocranium develops from mesodermal cells [23, 39–43], and only the nasal capsule and oropharyngeal skeleton develop from the cephalic (ANP and PHP, respectively) crest cells [24, 44]. In the lamprey, the trabecula (‘lamprey trabecula’) consists of a mesodermal neurocranial wall secondarily elongated rostrally [39–43]. In the hagfish, the corresponding prechondrogenic precursor later splits dorsoventrally into two bars: the dorsal longitudinal bar and ‘hagfish trabecula’ [23, 24]. Of these, the configuration of rostral elements of *Palaeospondylus* neurocranium agrees only with the hagfish-type, as the dorsal longitudinal bar and ‘hagfish trabecula’ occupy the corresponding position in this taxon.

**Fig. 4:**
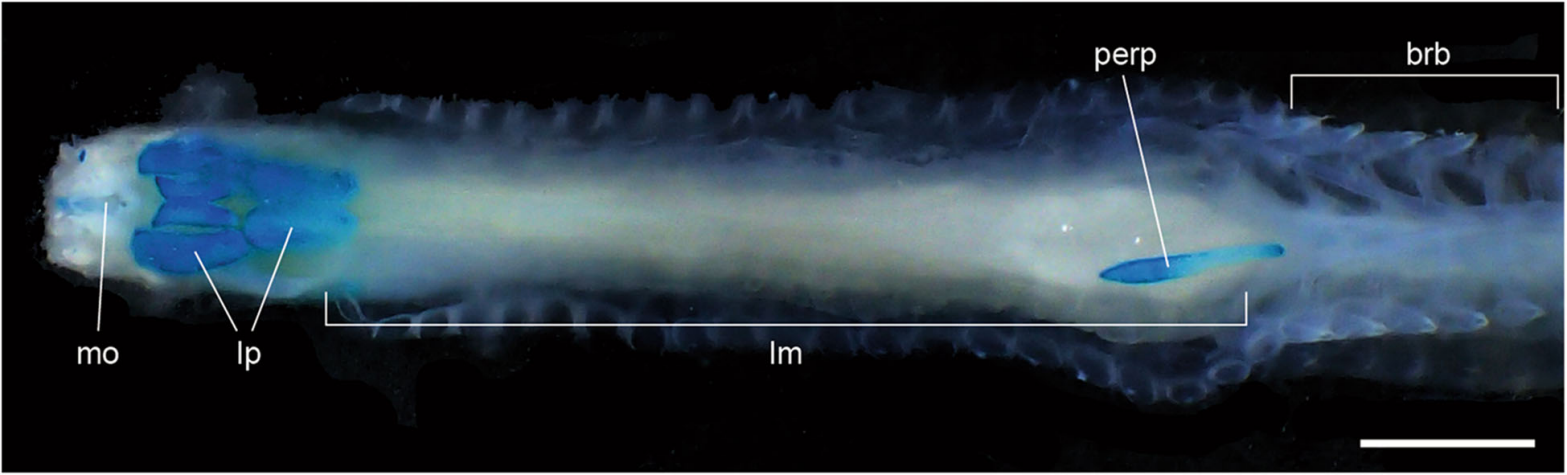
Alcian blue-stained specimen of the adult hagfish *Eptatretus burgeri* in ventral view. brb, branchial bascket; lm, lingual muscle (‘cyclostome tongue’); lp, lingual plate; mo, mouth; perp, perpendicular muscle cartilage (cartilage at the origin of the lingual muscle). Scale bar, 1 cm

One previous study argued against the cyclostome affinity of *Palaeospondylus*, citing the presence of a paired fin located caudally distant from the cranium [9]. The extant hagfishes, on the other hand, possess a cartilage at the origin of the lingual muscle (the ‘cyclostome tongue’ [45]), or the perpendicular muscle cartilage, which is located far caudal to the cranium [46] (Fig. 4). The perpendicular muscle cartilage of the extant hagfish is not decay-prone [47], and thus can be preserved separately from the cranial skeleton in fossils. Here, we suggest that the ‘paired fin’ of *Palaeospondylus* may in fact represent such a skeletal element at the origin of the lingual muscle.

Unique among known cyclostomes, *Palaeospondylus* possessed distinct features, including the biomineralization of cartilages and the well-developed vertebral column (notochordal centrum). These features, however, may have evolved from cyclostome-like conditions. The calcified cartilage of *Palaeospondylus* lacked perichondral ossification [48], which is specific to the gnathostomes [38], and it is conceivable that the calcified cartilage of *Palaeospondylus* evolved either through loss of perichondral ossification in gnathostomes, or through acquisition of biomineralization in cyclostomes. The latter possibility appears more plausible, as the hypertrophied cell lacunae in the cartilages of *Palaeospondylus* [11, 48] are reminiscent of cyclostome cartilages in their thin layers of extracellular matrix [21, 49]. A recent study also suggested that the vertebral element is synapomorphic to the vertebrates [21, 50], and the cylindrical vertebral column in *Palaeospondylus* (Additional file 1: Figure S1) may have evolved secondarily from arcualia in basal cyclostomes, through the invasion of cartilaginous cells into the fibrous sheath within the elastica externa [51]. The absence of arcualia in *Palaeospondylus* fossils does not preclude this scenario, since calcification often occurs differentially among vertebral elements, as seen in elasmobranchs [52].

There are nonetheless minor differences between *Palaeospondylus* and the known hagfish species, including the absence of some skeletal elements in *Palaeospondylus*. We suggest that this is likely due to a taphonomic bias. In the extant cyclostomes, there are two types of cartilage, hard and soft, which differ in the amount of extracellular matrix [49, 53, 54]. Soft cartilage is less resistant to decay than hard cartilage [47]. A similar heterogeneity of cartilage composition may have been present in *Palaeospondylus*, given that the branchial skeleton has not been identified in *Palaeospondylus*. The absence of branchial skeleton in *Palaeospondylus* fossils may indicate that cartilages of the branchial basket were not calcified, as in extant cyclostomes, for the functional reason that the branchial basket changes shape during ventilation. Among fossil cyclostomes, the Late Devonian putative stem-lamprey *Euphanerops* is a unique taxon that possessed calcified branchial basket [55]. The gill basket of *Euphanerops* was, however, unique in extending caudally across the half of the trunk, suggesting a functional requirement different from that in other cyclostomes.

In the extant hagfish chondrocranium, the rostralmost skeletal element made of hard cartilage is the subnasal cartilage (Fig. 2e). The subnasal cartilage is expected to be found rostral to the nasal capsule [47], but is apparently missing in *Palaeospondylus*. Since the proportion of the *Palaeospondylus* cranium resembles the embryonic, rather than the adult, cranium in known hagfish species (Fig. 2e), the absence of overt subnasal cartilage in *Palaeospondylus* fossils may reflect a very short snout containing a delicate subnasal cartilage. Cartilages supporting the nasal duct and tentacles in the extant hagfish are composed of soft cartilage [54], thus they are expected to decay rapidly.

**Fig. 5:**
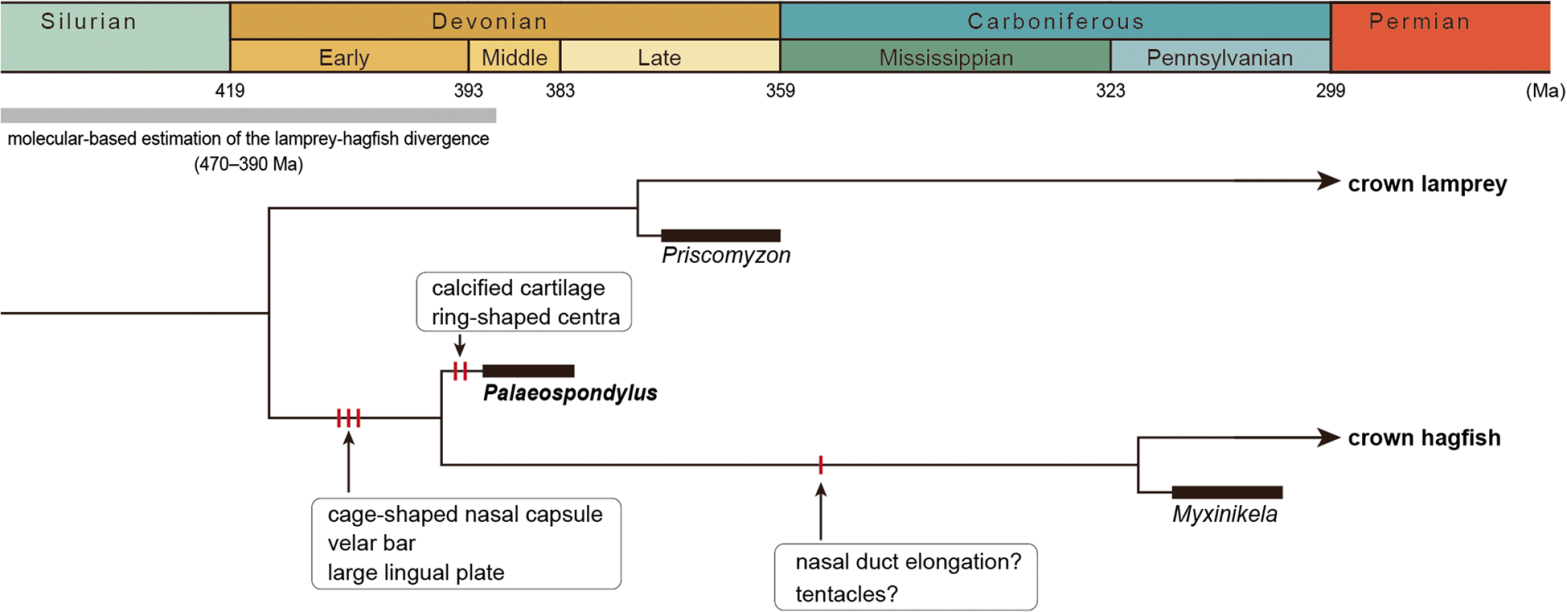
Proposed phylogenetic position of *Palaeospondylus*. The molecular-based estimation of the lamprey-hagfish divergence was adopted from [63]

Given the potential extensive taphonomic bias and the lack of data about soft tissue anatomy unlike circumstances of other species [56–58], it is difficult to build a character matrix to conduct a cladistic analysis of *Palaeospondylus*. Nevertheless, the above comparison strongly suggests that *Palaeospondylus* and the hagfish share a cranial skeletal configuration that is distinguishable from those of the lamprey and crown gnathostomes. On the other hand, some features seen in adult individuals of the known hagfish species are less conspicuous in *Palaeospondylus*. In particular, in the extant hagfishes, as well as in the Late Carboniferous hagfish *Myxinikela siroka* [59], the position of the nasal capsule, which initially develops at the rostral end of the cranium becomes relatively caudal in the cranium of adult individuals, whereas in *Palaeospondylus*, the nasal capsule remained at the rostral end. Based on this synapomorphy between extant hagfishes and *Myxinikela*, we suggest that the phylogenetic position of *Palaeospondylus* is best explained as a stem hagfish lineage basal to *Myxinikela* (Fig. 5).

The hagfish affinity of *Palaeospondylus* is important for the time of divergence between hagfish and lamprey lineages. The oldest reliable fossil record of the divergence time is the fossil lamprey *Priscomyzon riniensis* from the Upper Devonian (Famennian: 372–359 Ma in [60]) [61, 62]. An estimate using nucleotide and amino acid sequences, however, dated the divergence time to 470–390 Ma [63] (by the Middle Devonian), leaving a gap between the molecular-based estimate and the fossil record. Our classification of *Palaeospondylus* into the hagfishes pushes the oldest fossil record of the divergence time back to the Middle Devonian (393–383 Ma, Fig. 5), filling the gap between the molecular estimate and the fossil record, as well as depicting a hagfish species before the end-Devonian mass extinction that wiped out many vertebrate groups, including placoderms [64, 65].

Early cyclostomes may thus have been more morphologically diverse than previously recognized. At present, the early evolution of the cyclostomes has been less clear than that of the gnathostomes [62, 65]. Further analysis of *Palaeospondylus*, as well as other putative cyclostomes in the fossil record [55, 58, 66], may shed new light on the evolution of this lineage.

## Conclusions

We identified congruences in the arrangement of a suite of homologous skeletal parts between *Palaeospondylus* and the hagfish embryo, and conclude that *Palaeospondylus* was closely related to hagfishes. The gnathostome affinity of this species is improbable, since the topography of cranial skeletal elements of *Palaeospondylus* is consistent exclusively with the developmental craniofacial pattern of the cyclostomes. *Palaeospondylus* more closely resembles the embryo than the adult of the extant hagfish, suggesting that this species is placed at a stem hagfish position basal to the hitherto known hagfish species in the phylogeny.

## Additional files

Additional file 1: Figure S1. Fossils of *Palaeospondylus* from the Middle Devonian of the Scotland. (a) entire specimen of *Palaeospondylus gunni* (AMNH FF 10743) in ventral view. (b) cranial skeleton of *P. gunni* (AMNH FF 7586) in dorsal view. (c) Line drawing of B. (d) cranial skeleton of *P. gunni* (AMNH FF 10742) in ventral view. (e) Line drawing of D. amp, ampyx; cp, caudal plate; gam, gammation; hem, hemidome; otc, otic capsule; ros, rostralia; rp, rostral plate; ve, V-shaped element; vert, vertebra. Scale bar, 1 mm. (JPG 3 mb) Additional file 2: Figure S2. Referred *Palaeospondylus* specimens illustrated in previous studies. (a,b) A specimen in dorsal (a) and ventral (b) views, from [26]. (c,d) three-dimensional model reconstructed from sections in dorsal (c) and ventral (d) views, from [5]. (e) NHM (Natural History Museum, London) P 16123 in dorsal view, from [8]. (f), NHM P 16125 in ventral view, from [8]. amp, ampyx; cp, caudal plate; gam, gammation; hem, hemidome; otc, otic capsule; ros, rostralia; ve, V-shaped element; vert, vertebra. (JPG 860 kb) Additional file 3: Table S1. Comparison of craniofacial primordia in cyclostomes and crown gnathostomes. Based on [22]. (DOC 29 kb) Additional file 4: Table S2. Comparison of cranial skeletal elements among extant cyclostomes and *Palaeospondylus*. Names of skeletal elements in *Palaeospondylus* are shown by abbreviations employed in the present paper. For the nomenclature of the lamprey and hagfish chondrocrania, see [23]. (DOC 38 kb)
