# Supplementary figures and images for: Palaeospondylus as a primitive hagfish
Source: Zoological Lett. 2016 Sep 8;2(1):20. doi: 10.1186/s40851-016-0057-0 (PMC5015246; doi:10.1186/s40851-016-0057-0)

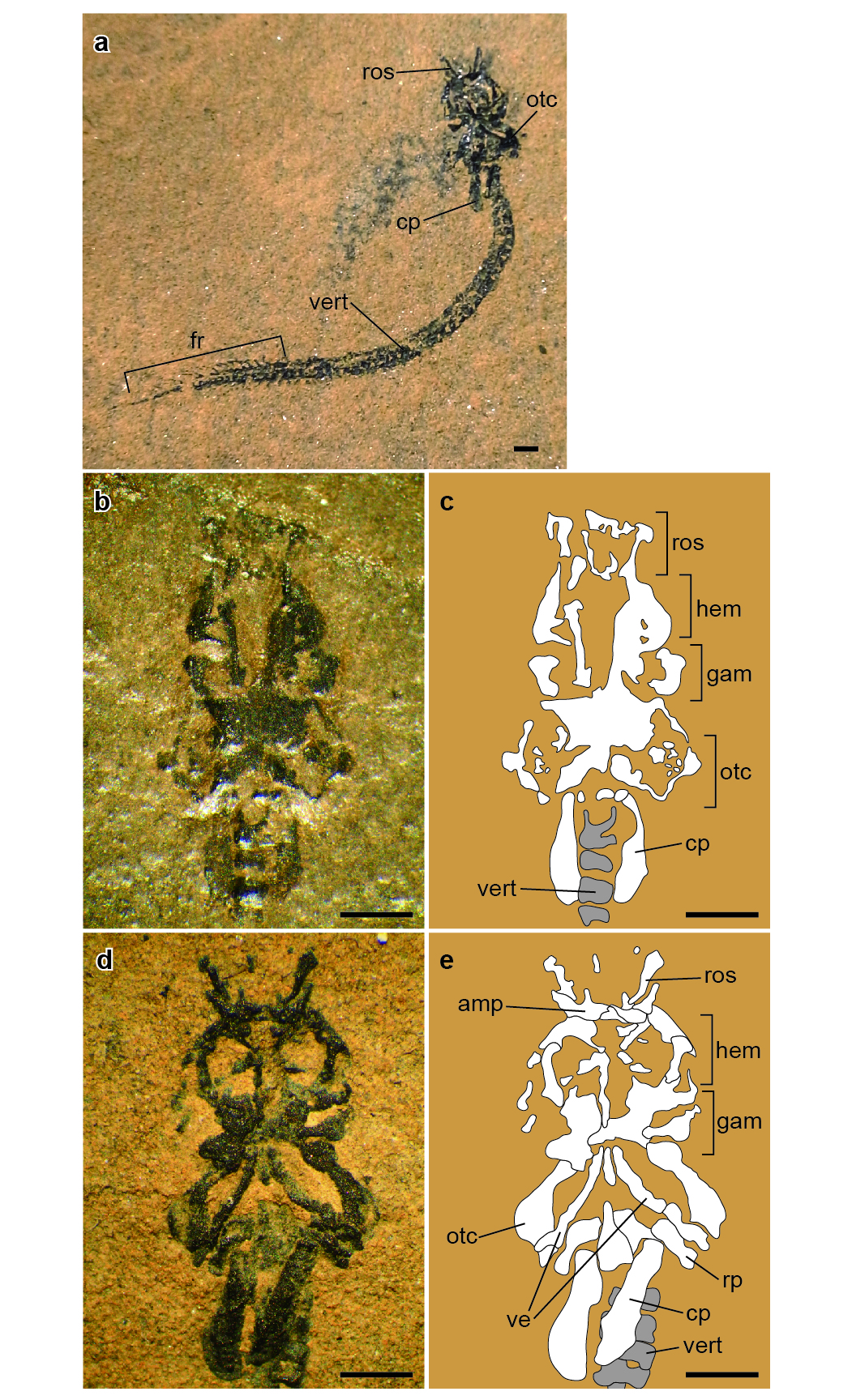

Supplement: Additional file 1: Figure S1. — Fossils of Palaeospondylus from the Middle Devonian of the Scotland. (a) entire specimen of Palaeospondylus gunni (AMNH FF 10743) in ventral view. (b) cranial skeleton of P. gunni (AMNH FF 7586) in dorsal view. (c) Line drawing of B. (d) cranial skeleton of P. gunni (AMNH FF 10742) in ventral view. (e) Line drawing of D. amp, ampyx; cp, caudal plate; gam, gammation; hem, hemidome; otc, otic capsule; ros, rostralia; rp, rostral plate; ve, V-shaped element; vert, vertebra. Scale bar, 1 mm. (JPG 3 mb) [file 40851_2016_57_MOESM1_ESM.jpg]

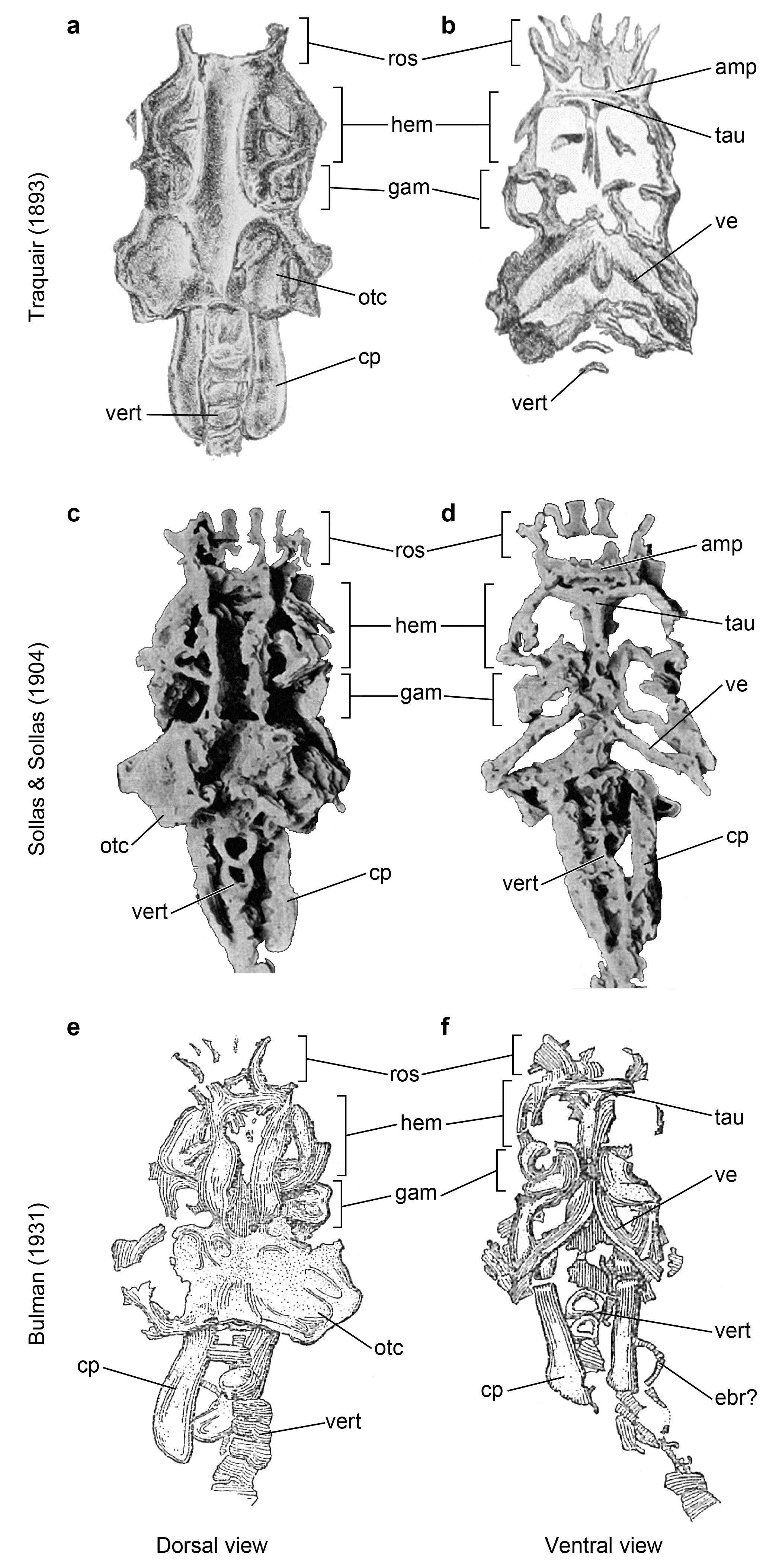

Supplement: Additional file 2: Figure S2. — Referred Palaeospondylus specimens illustrated in previous studies. (a,b) A specimen in dorsal (a) and ventral (b) views, from [26]. (c,d) three-dimensional model reconstructed from sections in dorsal (c) and ventral (d) views, from [5]. (e) NHM (Natural History Museum, London) P 16123 in dorsal view, from [8]. (f), NHM P 16125 in ventral view, from [8]. amp, ampyx; cp, caudal plate; gam, gammation; hem, hemidome; otc, otic capsule; ros, rostralia; ve, V-shaped element; vert, vertebra. (JPG 860 kb) [file 40851_2016_57_MOESM2_ESM.jpg]
