# Supplementary material for: Palaeospondylus as a primitive hagfish
Source: Zoological Lett. 2016 Sep 8;2(1):20. doi: 10.1186/s40851-016-0057-0 (PMC5015246; doi:10.1186/s40851-016-0057-0)
Supplement: Additional file 3: Table S1. — Comparison of craniofacial primordia in cyclostomes and crown gnathostomes. Based on [22]. (DOC 29 kb) [file 40851_2016_57_MOESM3_ESM.doc]

**Table S1. Comparison of craniofacial primordia between cyclostomes and crown gnathostomes.** Based on [21].

|  | cyclostomes | crown gnathostomes |
| --- | --- | --- |
| preotic crest cells | anterior nasal process (ANP) | lateral nasal prominence (LNP) |
|  |  | medial nasal prominence (MNP) |
| postoptic crest cells | posthypophyseal process (PHP) | postoptic ectomesenchyme (PO) |
| mandibular arch crest cells | mandibular arch (MA) | maxillary process (MX) |
|  |  | mandibular process (MN) |
