# Supplementary material for: Palaeospondylus as a primitive hagfish
Source: Zoological Lett. 2016 Sep 8;2(1):20. doi: 10.1186/s40851-016-0057-0 (PMC5015246; doi:10.1186/s40851-016-0057-0)
Supplement: Additional file 4: Table S2. — Comparison of cranial skeletal elements among extant cyclostomes and Palaeospondylus. Names of skeletal elements in Palaeospondylus are shown by abbreviations employed in the present paper. For the nomenclature of the lamprey and hagfish chondrocrania, see [23]. (DOC 38 kb) [file 40851_2016_57_MOESM4_ESM.doc]

**Table S2 |** **Comparison of cranial skeletal elements among extant cyclostomes and *Palaeospondylus*.** Names of skeletal elements in *Palaeospondylus* are shown by abbreviations employed in the present paper. For the nomenclature of the lamprey and hagfish chondrocrania, see [22].

| species  derivations | Hagfish | Lamprey | *Palaeospondylus* |
| --- | --- | --- | --- |
| ANP-derivatives | ant. vertical nasal bar post. vertical nasal bar nasal duct cart. | nasal capsule | avnb pvnb |
| PA1-derivatives | velar bar.  dors. longitud. bar (anterior)?  extra-mandibular (extrapalato-quadrate)  dental plate  linl plate  lat. basal cart. 1  med. basal cart. 1  third basal cart. | velar cart.  ventro-lateral plate (larva)  ventro-medial longitud. bar (larva)  dental plate (adult)  lingual cart. (adult)  ant. lateral apical cart. (adult)  medial apical cart. (adult)  piston cart. (adult) | vb  lp  dp |
| PHP-derivatives | rostral trabecula & trabecular commissure?  palatine bar (rostral connection of  longitudinal bar)  cornual cart.  labial cart.  subnasal cart. | trabecular commissure  lateral mouth plate (larva)  rostro-dorsal plate (larva)  styliform cartilage (adult)  post. lateral plate (adult)  post. dorsal plate (adult) ant. dorsal plate (adult) ant. lateral plate (adult) ant. dorsal plate (adult)  annular cart. (adult)  medio-ventral cart. (adult)  stylet cart. (adult) | palb  dorsal longitudinal bar  & its commissure  rostral trabecular bar |
| mesodermal neurocranium | otic caps. (part)  parachordals  posterior trabecula?  dorsal longitudinal bar (posterior)? | otic caps. (part)  parachordals  trabecula of the lamprey  subocular arch (adult) | otc  parachordals  posterior trabecular bar |
| PA2-derivatives | extrahyal | extrahyal | ?* |
| post. PA-derivatives | ext extrabranchiale 1  branchiale 1  extrabranchiale 2  branchiale 2 | branchiale 1  branchiale 2 | ?* |

* Putative extrabranchiale elements were described by [8].
